# Pharmacokinetic Comparisons of Typical Constituents in* Curcumae Rhizoma* and Vinegar-Processed* Curcumae Rhizoma* after Oral Administration to Rats

**DOI:** 10.1155/2018/6809497

**Published:** 2018-03-14

**Authors:** Wei Gu, Jin-ci Li, De Ji, Lin Li, Ji Zhang, Zi-hao Pan, Jun-jie Yang, Tu-lin Lu, Chun-qin Mao

**Affiliations:** ^1^School of Pharmacy, Nanjing University of Chinese Medicine, No. 138 Xian Lin Road, Nanjing 210023, China; ^2^Institute of Chinese Medicine, Yangtze River Pharmaceutical Group, 9 Xian Lin Road, Nanjing 210023, China; ^3^Xinyang College of Agriculture and Forestry, No. 49 The New 24 Street, Xinyang 464000, China

## Abstract

The Raw* Curcumae Rhizoma* (R-CR), included in the* Chinese Pharmacopoeia Edition 2015*, is a well-known Chinese herbal medicine. However, the vinegar-processed* Curcumae Rhizoma* (V-CR) is used more widely than R-CR. The pharmacokinetics comparison of R-CR and V-CR after oral administration to rats is poorly understood. A novel method, rapid resolution liquid chromatography-tandem mass spectrometry (RRLC-MS) coupled with a sensitive, specific, and convenient microdialysis sampling method, free from endogenous interference was developed in this research. The extracts of R-CR and V-CR were administered orally to each group of rats. The blood and liver microdialysis probes were positioned within the jugular vein toward the right atrium and the median lobe near the center of the liver, respectively. Then, a double-peak phenomenon was observed in the concentration-time curves of curdione in R-CR group, while it was not observed in V-CR group. The liver-to-blood distribution ratio of curdione in V-CR group increased significantly (*P* < 0.05) compared to that of R-CR group. However, compared with V-CR group, the pharmacokinetic parameters of curcumol exhibited no statistically significant differences from those of R-CR group. These results indicate that vinegar-processed procedure has influence on the pharmacokinetic process of* Curcumae Rhizoma* in/ns. RRLC*-*MS coupled with microdialysis system could be used to evaluate the pharmacokinetics of typical constituents in* Curcumae Rhizoma* after oral administration.

## 1. Introduction

The processing of traditional Chinese herbs, which is called “paozhi” in Chinese, is a conventional procedure before clinical medication is prescribed. Over a long history of thousands of years, it has been the key link between traditional Chinese medicine (TCM) and clinical application. That is to say, in order to meet different therapeutic requirements and/or reduce toxicity, the herbs could be specially treated before being used. There are many processing methods, which could be mainly divided into two categories, namely, the heating processing (stir-heating, steaming, boiling, roasting, etc.) and the compatible processing (processing with alcohol, vinegar, salt-water, ginger juice, honey, decoction liquid, etc.). The corresponding result of these processing methods is the change in property of Chinese medicine, which is the key of TCM processing principles. The change mainly refers to the orientation property and functional property that have been reflected from the clinical efficacy variety.

*Curcumae Rhizoma* (rhizome of Curcuma,* Ezhu *in Chinese) which belongs to the Zingiberaceae family is a widely used TCM over thousands of years. There are three species of* Curcumae Rhizoma* including* C. phaeocaulis*,* C. kwangsiensis,* and* C. wenyujin* used as* Ezhu*. Among them,* Curcuma wenyujin*, which is used in this study, is cultivated in southeast China, which has wide applications in pharmaceutical industry in China because of its wide biological activities. It has been frequently prescribed for the treatment of cardiovascular disease, inflammatory disease, and cancer, either alone or in combination with other herbs [1–4]. In order to achieve the liver targeting effect, R-CR is usually mixed with vinegar and boiled into black all over its inner and outer part, a process which was officially documented in the* Chinese Pharmacopoeia*. In addition, according to the* Chinese Pharmacopoeia Edition 2015*, there are more than fifteen Chinese patent drugs containing V-CR, such as Ertong-Qingre-Daozhi-Wan, Jiuqi-Niantong-Wan, and Xiao'er-Huashi-Wan. In clinical prescription, the V-CR is used more widely than raw pieces. Furthermore, it is reported that the R-CR has no significant effect on the cytochrome P450 isoforms of CYP1A2, CYP3A4, and CYP2E1, while the vinegar-processed pieces can inhibit CYP1A2 and CYP2E1 and have an inductive effect on the CYP3A4 [5].

For the modernization and globalization of TCMs, it is important to establish selective, sensitive, and feasible analytical methods for determination and quantification of bioactive components of TCMs in body fluids. At present, despite some reports on the pharmacological effects of R-CR and V-CR, little is known about their* in vivo* characteristics. Therefore, it is necessary to compare the difference of intracorporal processes of bioactive components in R-CR and V-CR, respectively. It is helpful for explaining the theoretic system of herbal channel-tropism.

As two kinds of sesquiterpenoids in the essential oil of* Curcumae Rhizoma*, curdione and curcumol are two major bioactive constituents commonly used as quality control markers [6, 7]. Both of them present a variety of pharmacological activities. Reports show that curdione may be a candidate for anti-inflammatory and cancer chemopreventive agents [8]. It also plays an important role in the inhibitory effect on platelet aggregation [9]. Curcumol has been reported to exhibit antihepatic fibrosis activity, possibly through the down-regulation of the transforming growth factor-*β*1 and cytochrome P450 in HSC-T6 cells [10]. In addition, it can induce apoptosis via caspases-independent mitochondrial pathway in human lung adenocarcinoma ASTC-a-1 cells [11]. To date, no assay has been reported for the comparison of* in vivo* distributions of curdione and curcumol in R-CR and V-CR, respectively.

Traditional biological sample pretreatment methods include protein precipitation with acid or organic solvent and liquid-liquid extraction followed by centrifugation. Microdialysis is a powerful sampling technique* in vivo* that enables the study of unbound tissue concentrations of both endogenous and exogenous compounds [12]. By inserting a probe into selected tissue or body fluid, microdialysis could offer information about substances directly at the site of action while being well tolerable and safe [13].

In this study, curdione and curcumol were selected as the target compounds to compare the* in vivo* characteristics, respectively, in R-CR and V-CR. An* in vivo* microdialysis system for simultaneous biological fluid sampling was established by inserting probes into the blood and liver of a rat, and then the microdialysates were analyzed by RRLC*-*ESI-MS. To the best of our knowledge, it was the first time to develop the established method for the comparison of pharmacokinetic characteristics of crude* Curcumae Rhizoma* and its processed products in rat microdialysis samples. In addition, this work could not only help to clarify the liver-target effect of V-CR, but also contribute to acknowledging the fact of “Chinese medicinal theory” and “the property of Chinese medicine changes after being processed.”

## 2. Methods

### 2.1. Chemicals and Reagents

Curdione (purity ≥ 98%) was purchased from Shanghai Huyun Medical Science Company (Shanghai, China), and curcumol (purity ≥ 98%) was obtained from the National Institute for Food and Drug control (Beijing, China). HPLC grade solvents and reagents were obtained from E. Merck (Darmstadt. Germany). Formic acid was purchased from Merck KGaA (Darmstadt, Germany). Triple deionized water from Millipore (Bedford, MA, USA) was used for all preparations. Anticoagulant citrate dextrose (ACD) solution (3.5 mM citric acid, 7.5 mM sodium citrate, and 13.6 mM dextrose) was prepared in our lab.

### 2.2. Plant Material

Fresh rhizome of Curcuma wenyujin were collected from Rui-an, Zhejiang, China, in December, 2015. The samples were identified as rhizome of Curcuma wenyujin by Professor Lu Tulin from Nanjing University of Chinese Medicine. Samples are divided into two parts on average and then processed into the Raw* Curcumae Rhizoma *(R-CR) and the vinegar-processed* Curcumae Rhizoma* (V-CR) according to the* Chinese Pharmacopoeia Edition 2015*, respectively.

### 2.3. Animals

Male Sprague-Dawley rats (250~300 g) were obtained from the Laboratory Animal Center of Nanjing University of Chinese Medicine. These animals were specifically pathogen-free and were housed in separate cages in a 12 h light-dark cycle at a temperature of 24 ± 1°C with the relative humidity of 55 ± 5 %, and they have free access to a standard diet and water. The animals were acclimatized for at least a week before any experimentation. Animal welfare and experimental procedures strictly conformed to the Guide for the Care and Use of Laboratory Animals (US National Research Council, 1996) and the related ethics regulations of Nanjing University of Chinese Medicine.

### 2.4. Preparation of Extraction of R-CR and V-CR

R-CR were collected from Yueqing, Zhejiang Province, China, and authenticated by Professor Jianwei Chen (Nanjing University of Chinese Medicine, JiangSu Province, China). R-CR was processed with vinegar to prepare V-CR according to the procedure recorded in* Chinese Pharmacopoeia* (National Commission of Chinese Pharmacopoeia, 2015).

The powdered herb materials of R-CR and V-CR were immersed into 90% ethanol for 1 h and then boiled with a reflux condenser 2 times for 1 h each time, respectively. The extracts were then merged and evaporated by rotary evaporation water vacuum at 40°C. Finally the samples were rediluted so that the concentration of R-CR and V-CR extract became the crude drug with the liquid density of 1.4 g/mL. The contents of curdione and curcumol in the extract were 0.21% and 0.079%, respectively, in R-CR and 0.18% and 0.043% in V-CR, respectively, which were determined by HPLC.

### 2.5. Preparation of Standard and Quality Control (QC) Samples

The standard stock solutions of curdione (0.53 mg/mL) and curcumol (0.65 mg/mL) were prepared in methanol. The combined standard working solutions were prepared by diluted ACD solution (citric acid: 3.5 mM; sodium citrate: 7.5 mM; dextrose: 13.6 mM) to reach concentrations of 3.33, 13.33, 26.65, 53.30, 106.60, and 213.20 ng/mL for curdione and 8.11, 32.45, 64.90, 129.80, 259.60, and 519.20 ng/mL for curcumol, respectively. For the validation of the method, QC samples were prepared in triplicate at the concentrations of 6.66, 26.65, and 191.88 ng/mL for curdione and 16.23, 64.90, and 467.28 ng/mL for curcumol by adding the blank microdialysate to the required amount of working stock cocktail solution in a volumetric flask.

### 2.6. *In Vivo* Microdialysis Experiments

Blood and liver microdialysis systems consisted of a microdialysis syringe pump (CMA/400, Stockholm, Sweden), a refrigerated fraction collector (CMA/470), microdialysis probes, and a heating pad. The probes for blood (10 mm in length) and liver (4 mm in length) were made of silica capillary in a concentric design and had a molecular weight cut-off of 15 kDa (Microbiotech/se AB, Stockholm, Sweden). When experimented, the rats were anesthetized with chloral hydrate solution (300 mg/kg, i.p.) and remained anesthetized throughout the experimental period. The blood and liver microdialysis probes were positioned within the jugular vein toward the right atrium and the median lobe near the center of the liver, respectively. The microdialysis systems were perfused with anticoagulant citrate dextrose, ACD solution at a flow rate of 2.0* μ*L/min. The rat temperature was maintained at 37°C with a heating pad during the experiment.

After a 2.0 h postsurgical stabilization period following the successful implantation of probes, the extract of R-CR and V-CR was given orally to each group of rats (14.17 g/kg of R-CR, consists of 29.77 mg/kg curdione and 11.20 mg/kg curcumol, and 14.17 g/kg of V-CR, consists of 25.50 mg/kg curdione and 6.10 mg/kg curcumol, respectively). The dialysate were collected every 30 min for each probe in a consecutive period of 8 hours. The collected samples were kept at −20°C and analyzed by RRLC*-*MS within 48 h.

### 2.7. High-Performance Liquid Chromatography and Tandem Mass Spectrometry Conditions

An Agilent 1200 series HPLC system (Agilent, USA) was equipped with an autosampler, a binary pump, and a thermostatically controlled apartment. Separation was achieved by an Agilent Zorbax SB C18 microbore column (2.1 × 50 mm, 1.8* μ*m). The column temperature was maintained at 30°C. The mobile phase consisted of Solvent A: acetonitrile-0.05% formic acid and Solvent B: water-0.05% formic acid by a gradient elution of 40%~45% A at 0~5 min, 45%~50% A at 5~8 min, 50%~55% A at 8~9 min, 55%~100% A at 9~10 min, and 100% A at 10~15 min. The flow rate was 0.4 mL/min. The injection volume was 20* μ*L.

Mass spectrometric detection was performed on a G6410B triple quadruple mass spectrometer (Agilent, USA) equipped with an electrospray ionization (ESI) source. The optimized parameters of the mass spectrometry were as follows: drying gas (N2), 8 L/min; drying gas temperature, 300°C; capillary temperature, 350°C; nebulizing gas pressure (N_2_), 35 psi; capillary voltage, 3000 V; the optimized potential (OP) for curdione and curcumol was 100 V and 70 V, respectively. Analysis was carried out in selected ion mode at the m/z 237.3 for curdione and m/z 219.2 for curcumol by Chemstation software (Agilent Technologies, USA).

### 2.8. Method Validation

For the pharmacokinetic study, blank blood and liver microdialysates for rats and blank ACD solution spiked with curdione and curcumol were evaluated for the peak interference.

The calibration curves of curdione and curcumol were constructed prior to the experiments using six standards ranging from 3.33~213.20 ng/mL of curdione and 8.11~519.20 ng/mL of curcumol with correlation coefficient of at least 0.995. The limit of detection was determined at a signal-to-noise ratio (S/N) of 3. And the limit of quantification was defined as S/N ratio of 10, which is the lowest concentration in the calibration curve in practice.

The intraday and interday precision for curdione and curcumol were determined by quantitating five replicates on the same day and in the following five consecutive days. The accuracy was calculated by comparing the average measured concentration (*C*_mea_) to the nominal concentration (*C*_nom_) and was expressed in percentage.

The stability of analytes was assessed by analyzing QC samples under three conditions, that is, freeze-thaw for three cycles, room temperature for 24 h, and storage in freezer at −20°C for 2 days.

The matrix effect was measured by comparing the peak area of the analytes dissolved in blank dialysates with those of pure standard solution containing equivalent amounts of curdione and curcumol.

### 2.9. Pharmacokinetic Analysis

Pharmacokinetic calculations were performed on each individual set of data with the pharmacokinetic program WinNonlin Standard Edition Version 5.2 (Pharsight, Mountain View, CA, USA) by noncompartmental method.

Statistics were analyzed by a SPSS16.0 program (SPSS Inc., Chicago, IL, USA). Comparisons between two groups were performed using the unpaired Student's *t*-*test*. The value of *P* < 0.05 was considered statistically significant. All results were expressed as arithmetic mean ± standard deviation (SD).

### 2.10. Establishment of HPLC Fingerprint of R-CR and V-CR

To establish the representative chromatographic fingerprint, extraction of R-CR and V-CR samples was analyzed using the optimized HPLC method as follows [14]. All separation was performed on an Agilent Series 1200 (Agilent Technologies, USA) liquid chromatograph, equipped with a binary pump, an autosampler, a vacuum degasser, an automatic thermostatic column compartment, and a DAD detector. A Hanbon ODS-2 C18 column (250 mm × 4.6 mm, 5* μ*m) was used for chromatographic separation at a column temperature of 30°C. The mobile phase was a mixture of acetonitrile containing 0.05% formic acid (mobile phase A) and water containing 0.05% formic acid (mobile phase B). The eluted gradient was as follows: 0–20 min, linear gradient 45–55% B; 20–40 min, linear gradient 55–75% B; 40–50 min, linear gradient 75–90% B; 50–65 min, linear gradient 90–100% B. The flow rate was 1.0 mL/min and the injection volume was 20* μ*L. The analytes were monitored with DAD at 216 nm and 430 nm [14].

## 3. Results

### 3.1. HPLC Fingerprint of R-CR and V-CR

**Figure 1:**
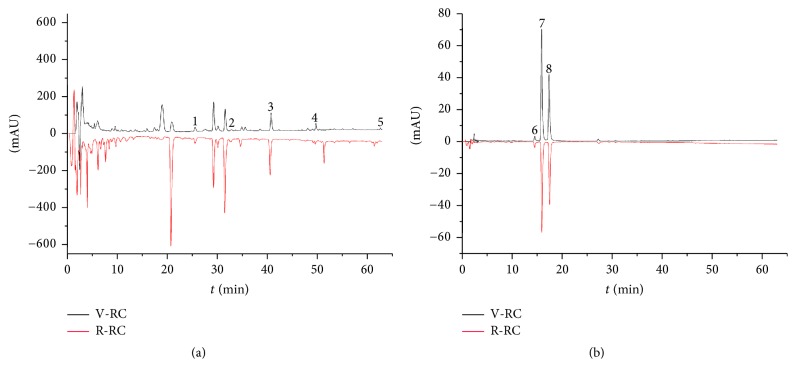
HPLC fingerprint of cruder and vinegar-processed* Curcumae Rhizoma* samples. (a) detection wavelength: 216 nm; (b) detection wavelength: 430 nm. HPLC conditions-column: Hanbon ODS-2 C18 column (250 mm × 4.6 mm, 5 *μ*m); mobile phase: (a) acetonitrile containing 0.05% formic acid, (b) water containing 0.05% formic acid; gradient program: 45–55% B (0–20 min), 55–75% B (20–40 min), 75–90% B (40–50 min), and 90–100% B (50–65 min); flow rate: 1.0 mL/min; temperature: 30°C; injection volume: 20 *μ*l. Five components monitored with DAD at 216 nm were identified as curdione (peak 1), curcumol (peak 2), germacrone (peak 3), furanodiene (peak 4), and *β*-elemene (peak 5); three components monitored at 430 nm were identified as bisdemethoxycurcumin (peak 6), demethoxycurcumin (peak 7), and curcumin (peak 8).

The validated two-wavelength HPLC method was used to establish HPLC fingerprint of R-CR and V-CR. The results are summarized in Figure 1. Among all the peaks, 8 peaks which are active components were chosen as the “characteristic fingerprint peaks” to represent the characteristics of* Curcumae Rhizoma*. Five components monitored with DAD at 216 nm were identified as curdione (peak 1), curcumol (peak 2), germacrone (peak 3), furanodiene (peak 4), and *β*-elemene (peak 5); three components monitored at 430 nm were identified as bisdemethoxycurcumin (peak 6), demethoxycurcumin (peak 7), and curcumin (peak 8) by comparing the retention time and UV spectrum of each peak with those of standard compounds (Table 1).

### 3.2. Method Validation

**Figure 2:**
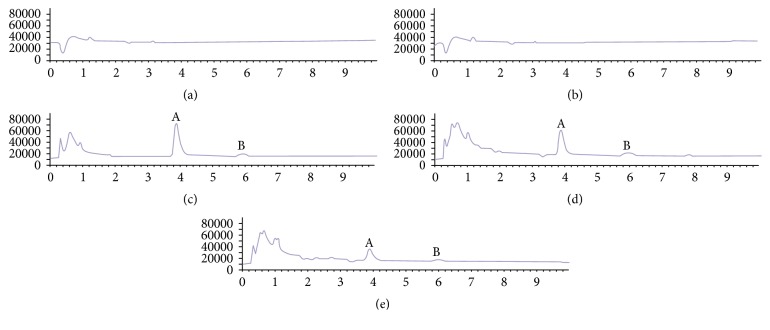
TIC chromatograms of curdione and curcumol ((a) blank blood microdialysate, (b) blank liver microdialysate, (c) ACD solution with reference standards of curdione and curcumol, (d) blood microdialysate, and (e) liver microdialysate; (A) curdione, (B) curcumol).

Representative chromatograms are shown in Figure 2, and no interference peaks from endogenous constituents were detected. Under the chromatographic conditions described, curdione and curcumol were eluted with the retention times of 3.9 min and 6.0 min, respectively.

Calibration curves showed excellent linearity over the concentration range from 3.33~213.20 ng/mL for curdione and 8.11~519.20 ng/mL for curcumol. Typical equations for the calibration curves were *Y* = 12285*X* − 1182.1 for curdione and *Y* = 962.56*X* − 3832.6 for curcumol. The lower limits of quantification (LLOQ) for curdione and curcumol were 3.33 ng/mL and 8.11 ng/mL, respectively.

The values for intraday and interday precision and accuracy for the QC samples are shown in Table 2. These results were within recommended limits by chemicals clinical pharmacokinetics technical guidelines and indicated that the established method was accurate, reliable, and reproductive.

Table 3 summarizes the stability data of QC samples. The results indicated that the analytical method offered satisfactory stability.

Studies of matrix effects of two analytes at QC concentrations gave concentrations within ±10% of nominal values (87.2~108.4% for curdione and 92.8~108.1% for curcumol), thus indicating that the matrix effects from salts and endogenous compounds could be negligible for the present method.

### 3.3. Recovery of Microdialysis Probes

The retrodialysis method was utilized to obtain the* in vivo* recovery for probe calibration. The blood and liver microdialysis probes were inserted into the jugular vein and the median lobe near the center of the liver under anaesthesia with chloral hydrate solution. ACD solution containing curdione (213.2 ng/mL) and curcumol (259.6 ng/mL) was injected into the rat blood and liver by the microdialysis probe at a constant flow rate of 2.0* μ*L/min. After a 2.0 h equilibration, the microdialysis samples were collected from each probe at the interval of 1.0 h and continued to collect for 6 h. The perfusate (*C*perf) and dialysate (*C*dial) concentrations of curdione and curcumol were determined by LC-MS. The* in vivo* relative recovery (*R*dial) was calculated according to the following equation:* R*dial = (*C*perf −* C*dial) /* C*perf. The average* in vivo* recovery of curdione and curcumol was 73.48 ± 4.70% and 66.09 ± 2.51% in rat blood and 76.33 ± 4.31% and 77.28 ± 3.89% in liver, respectively.

### 3.4. Pharmacokinetic Study

**Figure 3:**
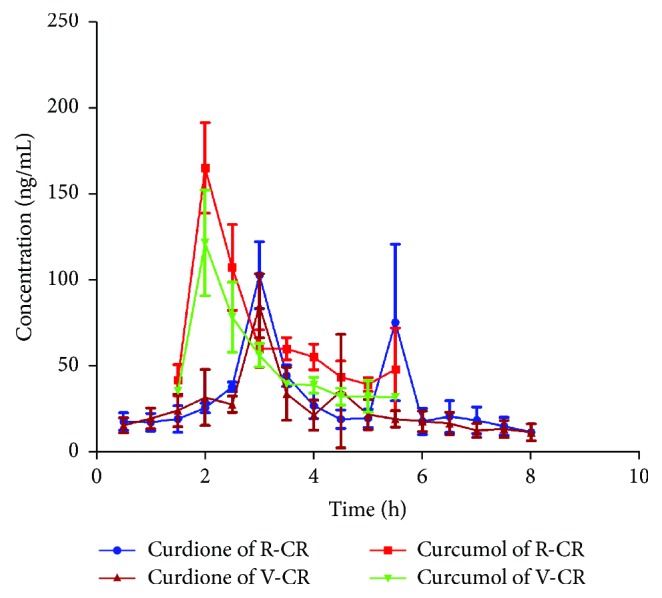
Blood concentration-time profiles of curdione and curcumol after oral administration of R-CR and V-CR.

**Figure 4:**
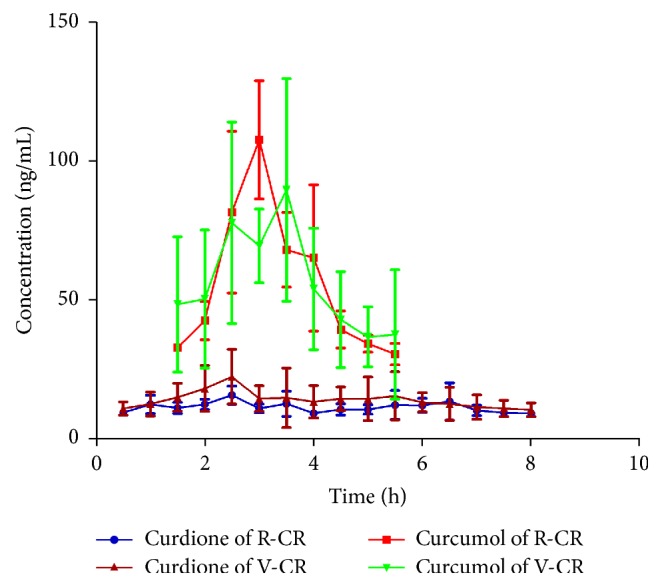
Liver concentration-time profiles of curdione and curcumol after oral administration of R-CR and V-CR.

The validated method was successfully applied to the pharmacokinetic study of curdione and curcumol in rat blood and liver microdialysates after oral administration of R-CR and V-CR extract. The mean concentration-time profiles are shown in Figures 3 and 4. The concentration-time data were analyzed by noncompartmental method and the pharmacokinetic parameters were summarized in Table 4.

#### 3.4.1. Pharmacokinetic Comparison of Curdione

As shown in Figure 3, about 3.0 h after oral administration of R-CR and V-CR extracts, the blood concentration of unbound curdione reached a maximum of 107.79 ± 18.86 ng/mL and 84.70 ± 18.55 ng/mL, respectively. It is interesting to notice that a double-peak phenomenon was observed in the concentration-time curves of curdione in R-CR group, while this kind of phenomenon is not obvious in V-CR group.

The pharmacokinetic parameters listed in Table 4 show that* C*max, AUC_0-*T*_, and AUC_0-*∞*_ of curdione in liver of V-CR group were 1.27-, 1.20-, and 1.10-fold greater than those of R-CR group, respectively. The significant increase in AUC suggests that curdione was absorbed better in the intragastric administration of V-CR extract than that of R-CR extract. In addition, the liver-to-blood distribution ratio of curdione calculated by dividing the AUC of curdione in liver by that in blood was 0.37  ±  0.08 for R-CR group and 0.55  ±  0.09 for V-CR group, which was considered a significant difference (*P* < 0.05) by statistical analysis.

#### 3.4.2. Pharmacokinetic Comparison of Curcumol

Figures 3 and 4 show the concentration-time profiles of curcumol in blood and liver of R-CR and V-CR group, respectively. The results revealed that curcumol was rapidly absorbed into the circulatory system and reached its peak concentration approximately 2 h after oral administration of R-CR extract. Moreover, the unbound curcumol in liver of R-CR group had a peak concentration at about 3.0 h and then was eliminated quickly. However, the profile of V-CR group shows that the blood and liver concentration of unbound curcumol maximized at about 2.9 h and 3.3 h, respectively. It is worth mentioning that a plateau occurs in liver concentration-time profile of V-CR group which maintains a period of high concentrations of unbound curcumol approximately from 2.5 h to 3.5 h.

Compared with the V-CR group, the pharmacokinetic parameters of curcumol exhibited no statistically significant differences from those of the R-CR group. Values of *C*_max_, AUC_0-*T*_, and AUC_0-*∞*_ of curcumol of V-CR group were marginally lower than those of R-CR group. The decrease of these three important parameters might result from the reduction of content in preparation of processed vinegar* Curcumae Rhizoma*.

## 4. Discussion

TCM is characterized by the processing of traditional Chinese herbs, which guides the diagnosis and prescription in clinic. R-CR and V-CR are two medication forms of* Curcumae Rhizoma* recorded in* Chinese Pharmacopoeia. *According to the processing theory of Chinese medicine, vinegar-processed procedure could alleviate the drug property and achieve the liver targeting effect.* Curcumae Rhizoma* is one of the most representative medicines requiring vinegar-processed step at present. Previous literatures have shown that R-CR is most effective at invigorating the circulation of Qi and driving stagnancy away, while V-CR works well in pain-alleviating, blood-activating and stasis-eliminating. Modern pharmacological studies so far have demonstrated that R-CR and V-CR can raise the pain threshold of mice and lower thrombocyte adherence rate of blood stasis model animals and the action of V-CR is more intense [15]. And V-CR shows better effect of prevention and treatment of hepatic fibrosis induced by CCl_4_ composited factor in rats [16]. In addition, studies on CYP450 activities showed that a long-term treatment of R-CR extract can inhibit CYP1A2 enzyme and induce CYP3A4 enzyme activities; however the V-CR extract has an effect of inhibiting CYP1A2 and CYP2E1 enzymes and inducing CYP3A4 enzyme activities both after single dose and multiple dose administration and the effect was in a time-dependent manner [5].

Many factors could contribute to the changes of pharmacological effects of* Curcumae Rhizoma* after being processed, like the changes of inherent chemical components, the alteration of* in vivo* pharmacokinetic processes, the herb-drug interactions, and so on. In terms of our results, the differences of pharmacological effects between R-CR and V-CR may be attributable to the pharmacokinetic and liver distribution changes of curdione and curcumol. The result of liver tissue analysis showed that the distribution of curdione of V-CR group was significantly different from R-CR group (*P* < 0.05). The concentrations of curdione in liver dialysates of V-CR group were evidently higher than those of R-CR group while concentrations of curdione in blood dialysates of V-CR group were declined compared to those of R-CR. It implied that vinegar-process procedure could alter a different drug distribution in liver and blood. Such result may partly explain the fact that vinegar-processed procedure could improve the bioavailability of curdione accounting for the liver targeting effect, while this phenomena was not observed in curcumol. The mechanism of increase efficiency after processed in Chinese medicine is extremely complex. But under the inspiration of curdione, it is assumed that if further improvement of detection method was achieved, more components might be observed targeting liver after oral administration of vinegar-processed CR, like curdione did. Determination and comparison of those main components could explain, at least to some extent, why this vinegar-process showed a better therapeutic effect. Moreover, the liver concentration-time profile of curcumol presented a long period of high concentration which may increase the therapeutic effect of curcumol.

Both curdione and curcumol belong to volatile compounds in* Curcumae Rhizoma* which possess various pharmacologic activities such as antimicrobial, antithrombotic, neuroprotective, anticancer, and antiviral activities. Previous studies showed that the contents of curdione and curcumol in V-CR decreased in varying degrees compared with raw pieces, which is consistent with our experiments. However, the* in vivo* experiments manifest a higher bioavailability of these two active ingredients in V-CR in comparison with R-CR. The probable reasons speculated according to literatures are the different influence on hepatocytes between V-CR and R-CR [10, 17, 18].

It is reported that the vinegar-processed* Radix Bupleuri* and* Rhizoma Rotundus* had significant influences on the permeability and the components of normal hepatic cell line of rats [19], which may directly affect the drug in the cell or cell-to-cell distribution. Thus it could be inferred that V-CR might also increase liver uptake of drugs through these approaches. However, further studies should be performed to clarify the hypotheses.

## 5. Conclusion

In this report, we delivered the first comparative pharmacokinetics study of curdione and curcumol in rats after intragastric administration of R-CR and V-CR extracts. A sensitive, specific, convenient, and endogenous interference-free microdialysis sampling method coupled with RRLC*-*MS was successfully developed and applied for the determination of protein-unbound curdione and curcumol in biological samples. There were statistically significant differences (*P* < 0.05) in the liver-to-blood distribution ratio of curdione between R-CR and V-CR groups. The obtained pharmacokinetic results indicated that vinegar-processed* Curcumae Rhizoma* could improve the bioavailability of curdione and curcumol, which may account for the liver targeting effect and better therapeutic effect of V-CR to some extent. This work would also contribute to the further development of TCM processing mechanism research and the reasonable application of Chinese herbal pieces in clinic.

## Figures and Tables

**Table 1:** The contents of each compound in crude and vinegar-processed *Curcumae Rhizoma *(*n* = 5).

Sample	Identified compounds and their mean content (mg/g)							
	Curdione	Curcumol	Germacrone	Furanodiene	*β*-Elemene	Bisdemethoxycurcumin	Demethoxycurcumin	Curcumin
R-CR	2.99 ± 0.11	1.13 ± 0.071	0.445 ± 0.038	0.133 ± 0.037	0.483 ± 0.061	0.0101 ± 0.0043	0.0475 ± 0.0078	0.0494 ± 0.0031
V-CR	2.10 ± 0.09	0.92 ± 0.082	0.356 ± 0.041	0.066 ± 0.012	0.376 ± 0.055	0.0046 ± 0.0029	0.0449 ± 0.0066	0.0476 ± 0.0045

**Table 2:** Intra- and interassay precision and accuracy of the method for the determination of curdione and curcumol (*n* = 5).

Analyte	Nominal (ng/mL)	Intraday			Interday		
		Found (ng/mL)	RSD (%)	Accuracy (%)	Found (ng/mL)	RSD (%)	Accuracy (%)
Curdione	6.66	7.83 ± 0.43	5.5	117.6	7.78 ± 0.64	8.3	116.9
	26.65	28.14 ± 2.70	9.6	105.6	29.44 ± 3.18	10.9	110.5
	191.88	197.22 ± 4.06	2.1	102.8	190.23 ± 12.72	6.7	99.1

Curcumol	16.23	18.99 ± 0.47	2.5	117.0	19.08 ± 0.88	4.7	117.6
	64.90	67.48 ± 5.23	7.8	103.9	67.65 ± 6.99	10.4	104.2
	467.28	513.81 ± 5.12	′1.0	110.0	472.08 ± 61.87	13.2	101.0

**Table 3:** Stability of curdione and curcumol in microdialysates (*n* = 5).

Analytes	Nominal concentration (ng/mL)	At room temperature for 24 h		Three freeze/thaw cycles		At −20°C for two days	
		Found (ng/mL)	RSD (%)	Found (ng/mL)	RSD (%)	Found (ng/mL)	RSD (%)
Curdione	6.66	7.94 ± 0.67	8.5	7.77 ± 0.60	7.8	7.67 ± 0.55	7.2
	26.65	28.46 ± 3.43	12.1	28.28 ± 2.82	10.0	29.17 ± 3.55	12.2
	191.88	194.17 ± 6.71	3.5	188.40 ± 10.8	5.8	189.1 ± 12.18	6.5

Curcumol	16.23	19.48 ± 0.60	3.1	19.49 ± 0.57	3.0	18.43 ± 0.93	5.1
	64.90	60.08 ± 6.69	11.2	63.79 ± 7.73	12.2	68.36 ± 5.35	7.9
	467.28	435.50 ± 3.45	0.80	464.66 ± 54.7	11.8	529.46 ± 75.2	14.3

**Table 4:** Pharmacokinetic parameters of curdione and curcumol in rat blood and liver after administration of R-CR and V-CR (*n* = 5).

Parameters	Blood					Liver					Blood-to-liver distribution
	*T*_max_ (h)	*C*_max_ (*μ*g/L)	AUC_0~*T*_ (*μ*g/L•h)	AUC_0~*∞*_ (*μ*g/L•h)	MRT_0~*T*_ (h)	*T*_max_ (h)	*C*_max_ (*μ*g/L)	AUC_0~*T*_ (*μ*g/L•h)	AUC_0~*∞*_ (*μ*g/L•h)	MRT_0~*T*_ (h)	AUC_liver_/AUC_blood_
R-CR											
Curdione	3.50 ± 1.12	107.79 ± 18.86	240.76 ± 49.53	269.71 ± 36.18	4.76 ± 0.65	4.00 ± 2.29	17.10 ± 5.13	87.61 ± 16.67	130.03 ± 18.53	7.24 ± 1.85	0.37 ± 0.08
Curcumol	2.00 ± 0.00	164.63 ± 26.23	312.17 ± 62.79	416.18 ± 96.25	3.89 ± 0.42	2.90 ± 0.22	111.67 ± 24.07	262.68 ± 53.00	400.91 ± 181.67	5.65 ± 3.80	0.85 ± 0.11
V-CR											
Curdione	3.00 ± 0.00	84.70 ± 18.55	199.86 ± 68.10	245.29 ± 79.99	5.28 ± 0.82	2.40 ± 0.22	22.53 ± 9.63	108.65 ± 43.75	145.98 ± 52.62	6.12 ± 0.64	0.55 ± 0.09^*∗*^
Curcumol	2.00 ± 0.00	135.14 ± 41.08	277.10 ± 103.13	369.83 ± 113.77	4.00 ± 1.10	3.30 ± 0.27	94.59 ± 36.89	240.53 ± 101.58	308.60 ± 117.57	3.92 ± 0.11	0.86 ± 0.09

^*∗*^ *P* < 0.05, unpaired *t*-test (two-sided) when value of V-CR group was compared to that of R-CR.
